# Diet and Mental Health: What Should Be Done for Malaysian Adolescents

**Published:** 2017-07

**Authors:** Esra TAJIK, Maryam JAVADI, Marjan MOHAMMADZADEH

**Affiliations:** 1. Dept. of Nutrition and Dietetics, Faculty of Medicine and Health Sciences, Universiti Putra Malaysia, Serdang, Selangor, Malaysia; 2. Dept. of Nutrition, School of Public Health, Qazvin University of Medical Sciences, Qazvin, Iran; 3. Children Growth Research Center, Qazvin University of Medical Sciences, Qazvin, Iran; 4. Dept. of Community Health, Faculty of Medicine and Health Sciences, Universiti Putra Malaysia, Serdang, Selangor, Malaysia

## Dear Editor-in-Chief

Nowadays, developing countries are in demographic, epidemiologic and nutrition transition leading to health pattern changes. The urbanization leads to psychosocial problems as well as unhealthy diet among adolescents (1). Unhealthy diet affects mental disorder and brain function through oxidative stress processes, inflammation and the stress-response system (2).

There are some evidence in Malaysia and other countries to reveal the relationship between diet and psychological factors in adults and adolescents. There is a reverse association between depressive disorder with traditional diet characterized by fruits and vegetables, fish, meat and whole grains, whereas the positive association with western diet of fried foods, sugary products, refined grains and beer (3). Similarly, Mediterranean dietary pattern has inverse relationship with depressive disorder (4), while western dietary pattern is positively associated with depressive disorder (5).

The association between food consumption and mental health is two-sided. However, food consumption can lead to stress, depression and mental disorder, people in stress situation or depressive mood has unhealthy food choices. Diet and food can promote mental health by adequate in-take of vitamins especially vitamin B, omega-3, minerals such as magnesium and calcium. While, food may damage mental health through skipping meals and consumption of foods, which produce oxidative, stress (6).

The components of healthy diet which can affect mental health includes whole grains, legumes, nuts, low-fat dairy, lean meats, olive oil, and fish, while the unhealthy diet includes consumption of white bread, fatty and processed meats (2). “Fruits and vegetables contain vitamins, antioxidants, beta-carotene and minerals and have been related to lower levels of markers of inflammation and oxidative stress” (7). “While fast food and junk food negatively affect the brain’s synapses and several molecules related to learning and memory” (5).

In addition, having main meals regularly are significant factors to show the quality of diet. Skipping main meals can lead to low diet quality and consequently affect the neural system (8). For example, breakfast consumption has a positive effect on cognitive performance and behavior of adolescents (9) and adolescents who used to skip breakfast had mental disorder (10).

**Fig. 1:**
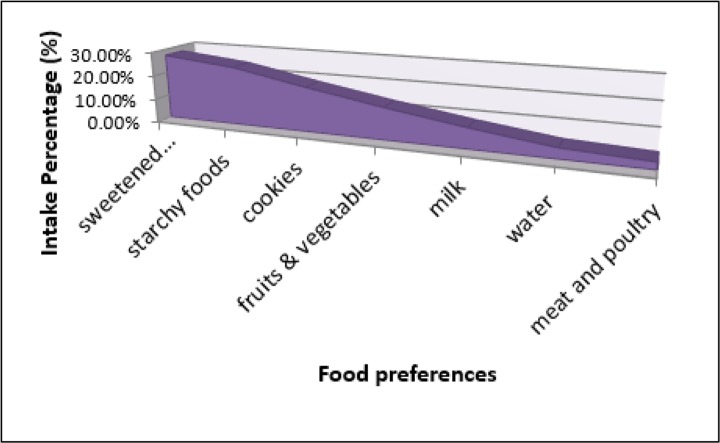
Food preferences among Malaysian adolescents

High prevalence of stress and depression among Malaysian adolescents urged researcher to do more studies on factors associated with mental health. Malaysian adolescents showed higher consumption of sweetened beverages (27.4%) and cookies (18.6%) than consumption of fruits and vegetables (13.3%), milk (8.5%) and water (4.4%) (Fig. 1). Moreover, 68% of them used to skip at least one of the main meals (10). They showed adolescents with higher unhealthy dietary behaviour had higher depression and/or stress symptoms from mild to extreme severe level (*P*<0.05).

As unhealthy diet (which leads to insufficient essential nutrient intake), has adverse health consequences, there is a need for implication of interventional studies and designing interventional packages for adolescents to improve healthy diet linked to mental health outcomes. The interventional packages should not only apply the healthy diet and psychological parameters, however, hire the motivational programs to improve the attitude of adolescents on reducing unhealthy diet. Health promoters and health educators should try to increase the knowledge on healthy diet and dietary behavior among adolescents. In this setting, the cooperation of families and schools (school principals, school canteen, and teachers) is necessary to reach the goal.

## References

[1] Shetty P (2013). Nutrition transition and its health outcomes. Indian J Pediatr, 80 Suppl 1: S21–7. 23412985 10.1007/s12098-013-0971-5

[2] Jacka FN Mykletun A Berk M Bjelland I Tell GS (2011). The association between habitual diet quality and the common mental disorders in community-dwelling adults: The Hordaland Health Study. Psychosom Med, 73(6):483–90. 21715296 10.1097/PSY.0b013e318222831a

[3] Jacka FN Pasco JA Mykletun A (2010). Association between western and traditional diets and depression and anxiety in women. Am J Psychiatry, 167(3):305–11. 20048020 10.1176/appi.ajp.2009.09060881

[4] Sánchez-Villegas A Henríquez-Sánchez P Ruiz-Canela M (2015). A longitudinal analysis of diet quality scores and the risk of incident depression in the SUN Project. BMC Med, 13:197. 26377327 10.1186/s12916-015-0428-y PMC4573281

[5] Akbaraly TN Brunner EJ Ferrie JE (2009). Dietary pattern and depressive symptoms in middle age. Br J Psychiatry, 195(5):408–13. 19880930 10.1192/bjp.bp.108.058925 PMC2801825

[6] Davison KM Kaplan BJ (2012). Nutrient intakes are correlated with overall psychiatric functioning in adults with mood disorders. Can J Psychiatry, 57(2):85–92. 22340148 10.1177/070674371205700205

[7] Baldrick FR Elborn JS Woodside JV (2012). Effect of fruit and vegetable intake on oxidative stress and inflammation in COPD: a randomised controlled trial. Eur Respir J, 39 (6):1377–84 22088966 10.1183/09031936.00086011

[8] Quirk SE Williams LJ O’Neil A (2013). The association between diet quality, dietary patterns and depression in adults: a systematic review. BMC Psychiatry, 13:175. 23802679 10.1186/1471-244X-13-175 PMC3706241

[9] Adolphus K Lawton CL Dye L (2013). The effects of breakfast on behavior and academic performance in children and adolescents. Front Hum Neurosci, 7:425. 23964220 10.3389/fnhum.2013.00425 PMC3737458

[10] Tajik E Latiffah AL Hamidin A (2016). Unhealthy diet practice and symptoms of stress and depression among adolescents in Pasir Gudang, Malaysia. Obes Res Clin Pract, 10(2):114–23. 26204813 10.1016/j.orcp.2015.06.001

